# Insights into microbiome and ARGs diversity in patients with upper and lower respiratory tract infections by targeted next-generation sequencing

**DOI:** 10.7717/peerj.21615

**Published:** 2026-08-31

**Authors:** Dongliang Wu, Tingyan Dong, Xiaoming Chen, Zetai Lin, Quanguan Pan, Jinhua Wei, Jinguang Liang, Jianxiang Wei

**Affiliations:** 1 Department of Pediatrics, Huangpu People’s Hospital of Zhongshan, Zhongshan, China; 2 Research Center for Healthy Aging and Social Economic Development, Guangzhou College of Commerce, Guangzhou, China; 3 Department of Pulmonary and Critical Care Medicine, Huangpu People’s Hospital of Zhongshan, Zhongshan, China

**Keywords:** Upper respiratory tract infections (URTIs), Lower respiratory tract infections(LRTIs), Respiratory microbes, Antimicrobial resistance gene (ARGs), Targeted next-generation sequencing (tNGS)

## Abstract

Respiratory tract infections (RTIs) cause substantial global morbidity and mortality, with antimicrobial resistance presenting an increasing challenge to the effective management. Characterizing the differences in microbiome composition and antimicrobial resistance genes (ARGs) between upper respiratory tract infections (URTIs) and lower respiratory tract infections (LRTIs) may inform site-specific diagnostic and therapeutic strategies. We retrospectively analyzed 1,340 URTIs samples (nasopharyngeal swab) and 699 LRTIs samples (bronchoalveolar lavage fluid) admitted to a single medical center to characterize the epidemiology of the respiratory microbes and ARGs using targeted next-generation sequencing (tNGS). Microbiome diversity, ARGs profiles, and coinfection patterns were compared between LRTIs and URTIs groups. Random forest machine learning was employed to identify discriminating species. LRTIs patients exhibited significantly higher microbiome abundance and ARGs diversity than URTIs patients (*P* < 0.001. Beta-lactam, multidrug, phenicol, and fluoroquinolone resistance genes were significantly more abundant in LRTIs (*P* < 0.01). Bacteria-virus coinfections predominated in both LRTIs (39.3%) and URTIs (54.6%). Thirty species were identified as potential discriminators between LRTIs and URTIs, with an Area Under Curve (AUC) of 0.852 in the training set. These findings reveal distinct microbial and ARGs profiles between URTIs and LRTIs patients, and provide a foundation for understanding site-specific microbial ecology in RTIs for clinical diagnosis and antimicrobial stewardship.

## Introduction

Respiratory tract infections (RTIs) rank among the leading causes of morbidity and mortality worldwide. The human respiratory tract harbors diverse microorganisms that form complex ecological communities essential for maintaining host physiological equilibrium (Zhao et al., 2023). These infections are anatomically classified as upper respiratory tract infections (URTIs), affecting the nasopharynx and oropharynx, or lower respiratory tract infections (LRTIs), involving the trachea, bronchi, and lung parenchyma. LRTIs typically confer greater severity and mortality risk than URTIs (Lynch 3rd & Kajon, 2021; Turhan et al., 2021). Previous investigations of respiratory microbiota have predominantly focused on isolated anatomical sites, either (Hurst et al., 2025) the upper respiratory tract or the lower respiratory tract (Han et al., 2025), or have examined specific patient populations such as those with chronic obstructive pulmonary disease or cystic fibrosis. Nevertheless, after the COVID-19 pandemic, research regarding the relationship between the microorganism communities in URTIs and LRTIs may yielded inconsistent findings. Comprehensive direct comparisons of microbiome composition and antimicrobial resistance genes (ARGs) profiles between URTIs and LRTIs within a unified cohort remain limited.

Antimicrobial resistance constitutes a critical global health threat (Padmanabhan, 2025). In RTIs management, empirical antibiotic therapy, often necessitated by delayed culture results, contributes to resistance selection (Adewusi et al., 2024). To develop effective and targeted treatment strategies for patients with RTIs, it is crucial not only to precisely identify a diverse range of pathogens but also to assess their resistance to antimicrobial agents (Haq et al., 2025). In numerous instances, evaluating ARGs is unfeasible because it first requires isolating a bacterial pathogen *via* culture prior to performing antimicrobial susceptibility testing (Ojeda et al., 2024). Traditional culture-based methods for pathogen and resistance detection are time-consuming and have low sensitivity, especially after prior antibiotic exposure (Adewusi et al., 2024; Lin et al., 2023). Metagenomic next-generation sequencing (mNGS) represents a revolutionary diagnostic approach that enables unbiased, high-throughput sequencing of nucleic acids extracted directly from clinical samples, independent of *in vitro* microbial culture. However, the high cost and complexity of bioinformatic analysis associated with mNGS limit its routine clinical application. As a new molecular diagnostic technology, targeted next-generation sequencing (tNGS) offers advantages such as detection sensitivity unaffected by human genome or background bacteria, lower costs, reduced sample transportation requirements, and the ability to quantitatively detect pathogens and ARGs (Ding et al., 2025).

In this study, we used tNGS to compare microbial and ARGs profiles between patients with URTIs and LRTIs in a large, retrospectively enrolled cohort. We compared the coinfection patterns across anatomical sites and identify microbial signatures capable of discriminating between URTIs and LRTIs using machine learning approaches. We hypothesized that LRTIs would demonstrate distinct microbiome profiles with enriched ARGs repertoires compared to URTIs, reflecting differences in microbial ecology and guiding the course of treatment.

## Material and methods

### Study design and patient population

This retrospective cohort study included patients admitted to Huangpu People’s Hospital of Zhongshan (Zhongshan, China) from April 2024 to April 2025. The study was approved by the Ethics Committee of Huangpu People’s Hospital of Zhongshan (number:hpyy-wwlw-2025-002), and all participants provided written informed consent.

Patients were classified into URTIs (*n* = 1,340, nasopharyngeal swabs) or LRTIs (*n* = 699, bronchoalveolar lavage fluid (BALF)) groups based on final clinical diagnosis established through composite reference standards including clinical manifestations, microbiological evidences, imaging findings, and clinical adjudication by attending physicians. The inclusion criteria were: (1) patients presenting with clinical symptoms and signs suggestive of RTIs, such as cough, fever, and sputum production; (2) patients with radiological evidence of RTIs, such as pulmonary infiltrates on chest X-ray or computed tomography; (3) patients with positive culture results from respiratory specimens (*e.g.*, sputum, bronchoalveolar lavage fluid, or nasopharyngeal swabs); and (4) patients who provided written informed consent for the tNGS examination. The exclusion criteria were: (1) patients who declined the tNGS examination; (2) samples failing quality control during tNGS processing; and (3) patients with incomplete clinical or laboratory data. The distinct clinical criteria for each group reflect fundamental differences in diagnostic approaches: URTIs diagnosis relies primarily on clinical examination and symptomatology, whereas LRTIs requires radiological confirmation of parenchymal involvement. These diagnostic differences inherently influence microbiological interpretation, as BALF sampling represents a more invasive procedure typically reserved for severe or complicated cases, potentially selecting for more complex microbiomes.

### Nucleic acid extraction and library preparation

According to the manufacturer’s instructions for the ZymoBIOMICS™ DNA Miniprep Kit (Zymo Research, R2002), at least one nasopharyngeal swab and 5ml of BALF were separately collected for DNA extraction. Extracted nucleic acids were quantified and subjected to polymerase chain reaction (PCR) amplification. Multiplex PCR-based targeted sequencing was used to detect predefined respiratory pathogens. In the PCR assay, a panel of pathogen-specific primers were used together with internal control primers. The thermal cycling protocol consisted of: (i) an initial denaturation at 95 °C for 3 min; (ii) 25 cycles of denaturation at 95 °C for 20 s, annealing for 20 s, and extension at 60 °C for 4.5 min; and (iii) a final extension at 72 °C for 5 min. Amplified products were purified using magnetic beads. Subsequently, targeted capture and library construction were performed. Qualified DNA libraries were sequenced on the Illumina high-throughput sequencing platform. Sequencing data were automatically normalized according to the amplification calibration coefficient. Reads shorter than 60 bp or exhibiting non-specific primer binding were filtered out; the remaining clean reads were used for bioinformatics analysis.

### Targeted next-generation sequencing analysis

For diagnostic purposes, the identification panel comprised 199 pathogens (Table S1). Initially, DNA sequences were chosen as the targeted fragments. Multiplex PCR was employed to amplify and enrich these targeted gene sequences. Sequencing adaptors were ligated to the purified PCR products, which were then purified again using magnetic beads. Targeted sequencing (300 cycle paired-end) was conducted on an MGI200 system under the FGS-SE60 test mode. Raw sequencing data were processed using MiSeq Reporter software (BWA, 0.7.17-r1188) to generate FASTQ files. The offline data were identified and counted, and reads with a double-end length greater than 60 bp were retained.Reads exhibiting low complexity, single-end primer recognition, or non-specific primer binding were filtered out. The resulting high-quality read pairs constituted the clean reads used for for identification and sequence alignment. Read pairs that aligned with the human genome were discarded to minimize host DNA contamination. The remaining read pairs were subsequently aligned to microbes reference sequences. Read paired counts for each microbes were generated for further analysis.

### Analysis of antimicrobial resistance genes (ARGs)

All non-human source reads were searched for ARGs using Structured Antibiotic Resistance Genes (SARG) database using UBLAST, with an *E*-value threshold of ≤10^−7^. The SARG database integrates annotations from ARDB, CARD, and the NCBI RefSeq database (Lu et al., 2022). ARGs identification required a minimum alignment length of 75 nucleotides and a nucleotide identity of ≥80%.To enable cross-specimen comparison of ARGs abundance across BALF samples, ARGs counts were normalized to copies per microbial cell using the formula described in a previous study (Chen et al., 2023). The heatmap for ARGs was created using self-developed R scripts.

### Random forest is used to identify representative species among groups

To identify potential species that could be used to distinguish between URTIs and LRTIs, we constructed a random forest model using the R package “RandomForest” (Version 4.7) (Candek, Pristovsek Candek & Kuntner, 2020). The combined dataset of the URTIs and LRTIs group was randomly partitioned into the training and testing set with a ratio of 1:1. The number of trees (ntree) was set to 500 after confirming that out-of-bag (OOB) error stabilized beyond this point. The number of variables randomly sampled as candidates at each split (mtry) was set to the square root of the total number of predictor variables (default setting for classification). All other parameters were kept at default values. Ten-fold cross-validation was performed on the training set to evaluate model stability and reduce overfitting. OOB error rates were monitored throughout. Feature selection was conducted exclusively on the training set using the mean decrease accuracy (MDA) metric, which quantifies the reduction in classification accuracy when a given variable is permuted. Species were ranked by MDA. A threshold of MDA >3.5 was applied to select the most discriminatory species, based on the distribution of importance scores and cross-validated performance. This threshold ensured that only features with a meaningful contribution to classification accuracy were retained. Model performance was evaluated using receiver operating characteristic (ROC) curve and area under the curve (AUC) calculations with the R package “pROC” (Version 1.18) (Ardila, Gonzalez-Arroyave & Tobon, 2025).

### Statistical analysis

Statistical significance was defined as *p* < 0.05 using two-tailed hypothesis tests. Group comparisons were performed using the Wilcoxon rank sum test for binary variables continuous variables and Fisher’s exact test for binary variables. Principal component analysis (PCA) was performed using the prcomp() function (R stats package). All statistical analyses were conducted in R (version 4.1.2).

## Results

### Microbial diversity discrepancies in LRTIs and URTIs patients

**Figure 1:**
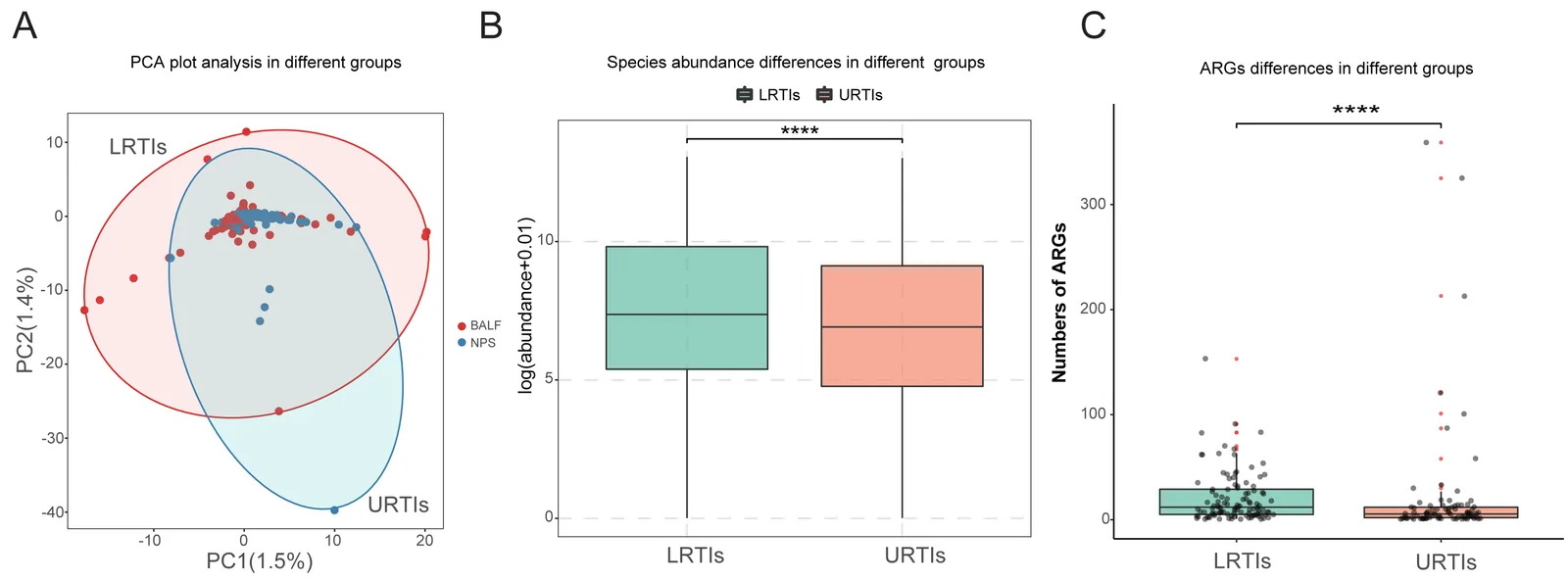
Differences in composition of microbiomes and ARGs between LRTIs and URTIs patients. (A) Principal component analysis (PCA) analysis was performed to identify the primary direction of variation in species composition across samples. (B) Differential species abundance analysis between LRTIs and URTIs patients through log(abundance + 0.01) transformation. (C) Comparison of ARGs abundance between LRTIs and URTIs patients. The levels of significance for the Wilcoxon rank-sum test are: **** for *P* < 0.001.

All known microbial species were screened, and a relative abundance matrix of pathogens was constructed. PCA plots showed that microbial community structure in the LRTIs and URTIs group gathered into a similar cluster on the projection plot (Fig. 1A). Differences in pathogen abundance across sample types were analysed through log-transformed abundance values (log(abundance + 0.01)) and the Wilsons rank-sum test. Significant differences were observed between the RTI microbiome profiles of LRTIs and URTIs patients (Fig. 1B, *P* < 0.001). Total ARGs counts were also analysed using the Wilcoxon rank-sum test, revealing significantly higher ARGs numbers in LRTIs samples than in URTIs samples (Fig. 1C, *P* < 0.001). These results indicate greater microbial and ARGs diversity in LRTIs patients compared with URTIs patients.

### Comparative analysis of microbiomes variations in LRTIs and URTIs patients

**Figure 2:**
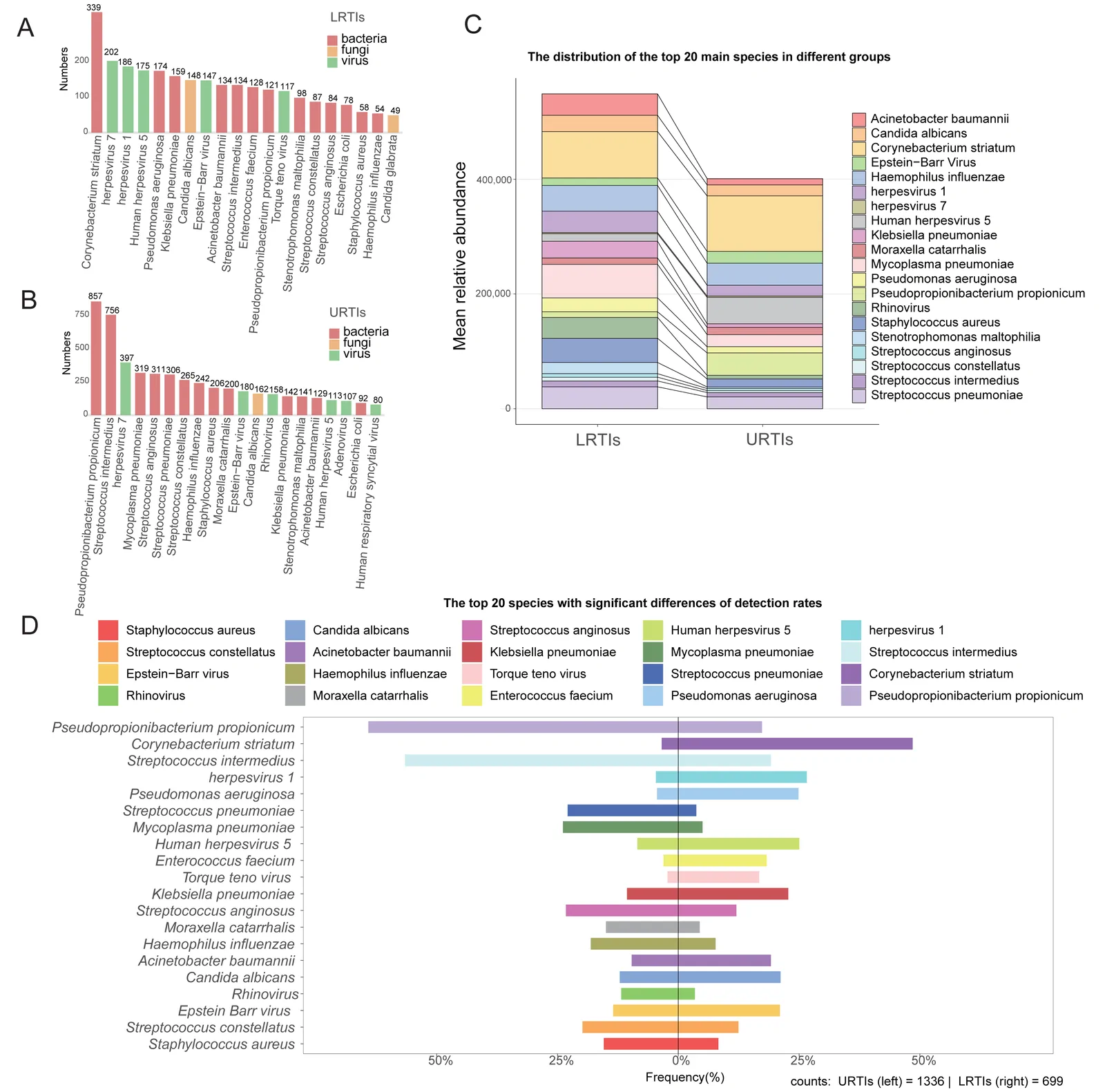
Distribution of pathogens in (A) LRTIs and (B) URTIs patients (C) Bar chart showing the top 20 common species between LRTIs and URTIs patients. Each color bar represents one species. (D) Frequency distribution of the top 20 significantly difference species detected in RTIs patients, stratified by infection type: LRTIs (right) and URTIs (left). Species are indicated by color (*n* = 2039 patients).

The top 20 most frequently detected pathogens were subjected to statistical analysis. *Corynebacterium striatum*, human herpesvirus 7 and human herpesvirus 1 in LRTIs patients (Fig. 2A), whereas *Streptococcus intermedius*, human herpesvirus 7 and *Mycoplasma pneumoniae* were most common in URTIs patients (Fig. 2B). The top 20 main species were distributed between the LRTIs and URTIs patients, especially enriched with *Corynebacterium striatum*, herpesvirus 1, *Haemophilus influenzae*, *Mycoplasma pneumoniae*, *Staphylococcus aureus* and *Streptococcus pneumoniae* (Fig. 2C). Meanwhile, the variance analysis calculated significantly difference species. It was showed that *Corynebacterium striatum*, herpesvirus 1, *Pseudomonas aeruginosa*, Human herpesvirus 5, *Enterococcus faecium*, Torque teno virus, *Klebsiella pneumoniae*, *Acinetobacter baumannii*, *Candida albicans*, and Epstein Barr virus were significantly abundant in LRTIs patients than that in the URTIs patients. However, Streptococcus *pneumoniae, Mycoplasma pneumoniae, Streptococcus anginosus*, *Moraxella catarrhalis*, *Haemophilus influenzae*, *Rhinovirus* and *Streptococcus constellatus* in the URTIs patients were significantly enriched compared to the LRTIs patients (Fig. 2D). The species detected in each sample were listed in Table S1.

### Comparative analysis of ARGs variations in LRTIs and URTIs patients

**Figure 3:**
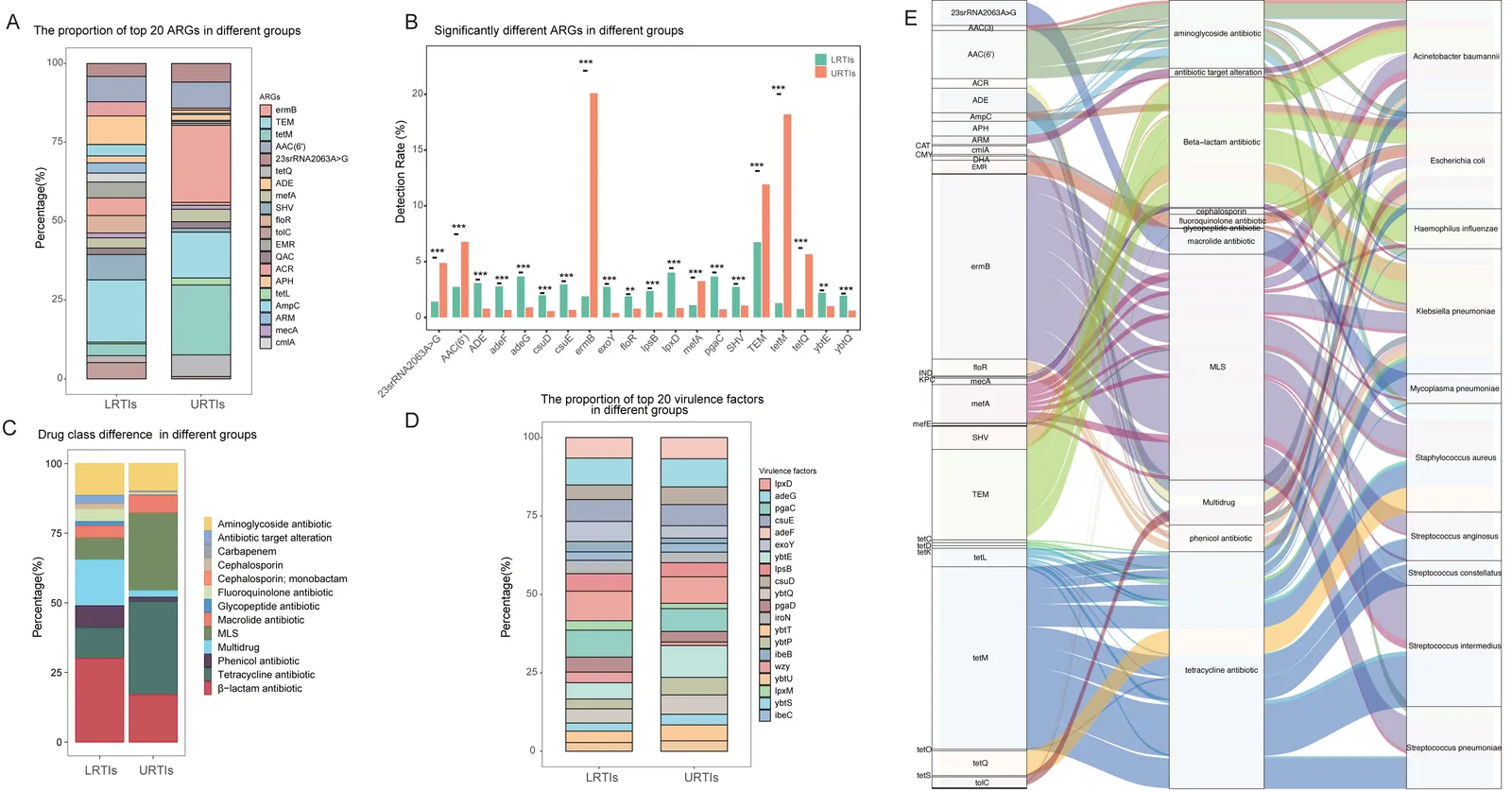
ARGs and antibiotic resistance analysis. (A) Cumulative bar plot showing the distribution of the top 20 antibiotic resistance types between LRTIs and URTIs patients. (B) Significantly difference of ARGs and virulence factors in LRTIs and URTIs patients. The level of significance for the Wilcoxon rank-sum test are: ** for *P* < 0.01; ***: *P* < 0.001. (C) Comparison of the abundance of antibiotic classes across groups. (D) The cumulative bar graph represents the distribution of the top 20 virulence factors in different groups. (E) The Sankey diagram visualizes the associations between pathogens and their corresponding ARGs, as identified through targeted tNGS. Each color represents a distinct bacterial species and its associated antimicrobial resistance profile.

The top 20 most abundant ARGs differed significantly between LRTIs and URTIs patients. Among LRTIs patients, TEM, ADE, SHV, AAC(6″), and ermB were the 5 most frequently detected ARGs, whereas URTIs showed predominance of ermB, tetM, TEM, AAC(6″), and 23srRNA2063A>G (Fig. 3A). Significant differences in the abundance of these ARGs were observed between the two patient groups (Fig. 3B). Age-stratified analysis revealed that these differences persisted significantly across the 0–17 years, 40–64 years, and 65–99 years age groups, but not in the 18–39 years group (Fig. S1A). Furthermore, sex-stratified analysis demonstrated that overall ARGs abundance was significantly higher in LRTI patients than in URTI patients, consistently in both males and females (Fig. S1B).

Antibiotic class-level analysis revealed higher abundance of beta-lactam antibiotic, multidrug, phenicol antibiotic, fluoroquinolone, antibiotic target alteration in LRTIs patients. Conversely, tetracycline antibiotic, macrolide–lincosamide–streptogramin (MLS) and Carbapenem were more abundant in URTIs patients (Fig. 3C). These correlations may reflect differences in antibiotic exposure patterns, bacterial community composition, or patient population characteristics between groups, though causal relationships cannot be established.

Virulence factor analysis showed adeF, adeG, csuD, csuE, exoY, lpsB, lpxD, pgaC, ybtE and ybtQ were significantly more abundant in LRTIs patients (*P* < 0.01) (Fig. 3B). lpxD, adeG, adeF, pgaC, and lpsB, were the top five most abundant virulence factors in both LRTIs and URTIs patients (Fig. 3D). Correlation analysis revealed positive associations between specific pathogens and ARGs classes. For example, *Acinetobacter baumannii* correlated with beta-lactam and multidrug resistance genes, while *Streptococcus pneumoniae* correlated with MLS resistance (Fig. 3E). In addition, abbreviations of antibiotic types, ARGs in each sample, and other categories of drugs and resistance mechanisms were listed in Table S2.

### Coinfection analysis between LRTIs and URTIs patients

**Figure 4:**
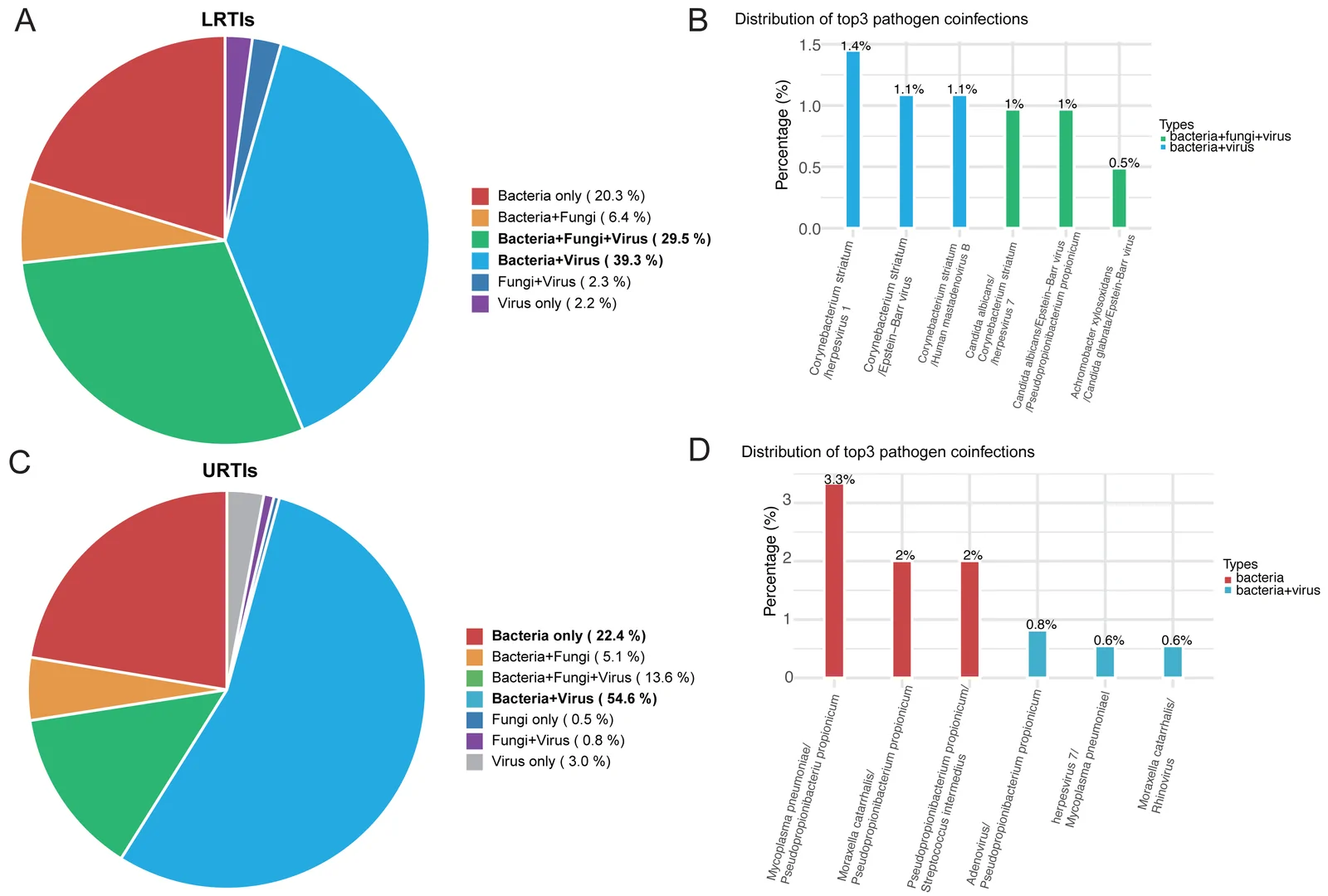
Coinfection analysis between LRTIs and URTIs patients. (A) Pie chart showing microbiota composition in cases of mixed respiratory tract infection among LRTI patients. (B) Pie chart showing the top three pathogens identified by tNGS in bacterial–viral and bacterial–fungal–viral coinfections among LRTI patients. (C) Pie chart showing microbiota composition in cases of mixed respiratory tract infection among URTI patients. (D) Pie chart showing the top three pathogens identified by tNGS in bacterial and bacterial–viral coinfections among URTI patients.

Bacterial-virus coinfection represented the most frequent pattern in both LRTIs patients (39.3%) and URTIs patients (54.6%). In addition, bacteria-fungi-virus coinfections were notably more common in LRTIs patients (29.5%) than URTIs (13.6%), whereas bacteria-only infections predominated in URTIs patients (22.4%) (Figs. 4A, 4C). Among the most frequently coinfection types, the top three pathogens were analysed. *Corynebacterium striatum*, Candida albicans and Epstein-Barr virus were always involved coinfections with other pathogens in LRTIs patients (Fig. 4B), Meanwhile, in URTIs patients, the coinfections type tend to be single-species (Fig. 4D), which was different from the multi-species in LRTIs patients.

### Identification of potential microbes for discriminating LRTIs and URTIs

**Figure 5:**
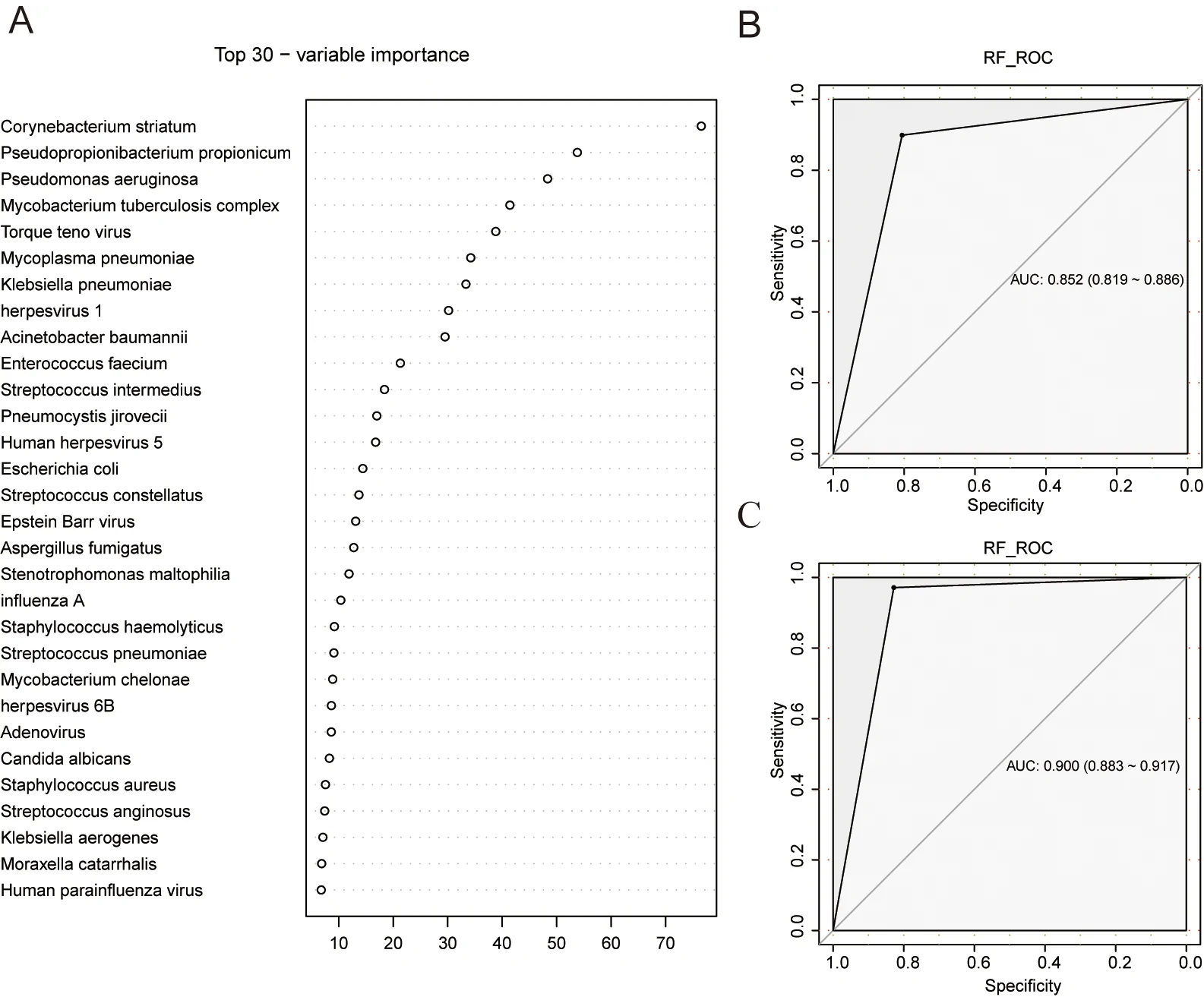
Identification of important species for discriminating LRTIs from URTIs patients. (A) Histogram showing the variable importance scores of the top 30 microbial species in the Random Forest model. (B) Receiver operating characteristic (ROC) curve for the Random Forest supervised machine learning model trained on the training set. (C) ROC curve for the Random Forest supervised machine learning model evaluated on the testing set.

To identify microbial signatures distinguishing infection sites beyond descriptive comparisons, we applied a Random Forest supervised learning model. The diagnostic power of the identified biomarker panels was further evaluated by receiver-operating characteristic (ROC) analysis. We selected species with a mean decrease accuracy >3.5 to identify the most important features for discrimination. This approach allowed us to rank pathogens by their contribution to classification accuracy. Finally, 30 representative species (Fig. 5A) were identified, and performance assessment of the set presented high classification efficiency, with an AUC of 0.852 in the training set (Fig. 5B) and 0.900 in the testing set (Fig. 5B). Thus, these 30 species show strong potential for discriminating LRTIs from URTIs patients. However, these performance metrics reflect technical discrimination capacity within this specific cohort and do not establish clinical diagnostic utility, which would require prospective validation with healthy controls, diverse clinical presentations, and outcome assessment.

## Discussion

A diverse and complex community of microorganisms inhabits the human respiratory tract (Natalini, Singh & Segal, 2023). Even though numerous studies employing culture-independent metagenomic analyses have been carried out to evaluate the microbiome composition at different sites of the human respiratory tract (Mancabelli et al., 2022), a comprehensive comparative analysis of URTIs and LRTIs patients is still absent. This large-scale retrospective study directly compares respiratory microbiome composition and ARGs profiles between URTIs and LRTIs patients based on the tNGS methodology. Our findings demonstrate distinct microbial community structures and resistance gene patterns between anatomical sites, with LRTIs showing greater diversity and enrichment of healthcare-associated pathogens and resistance determinants.

In this study, we aimed to investigate differences in microbiome spectra between patients with URTIs and LRTIs. In this research, bacterial species like *Corynebacterium striatum*, *Streptococcus pneumoniae*, *Streptococcus intermedius*, *Streptococcus anginosus*, *Moraxilla catarrhalis*, *Haemophilus influenza* and *Klebsiella pneumoniae* were frequently identified. Meanwhile, the findings of *Streptococcus pneumoniae*, *Staphylococcus aureus* and *Moraxilla catarrhalis* enriched in the URTIs patients, which were consistent with a previous study that they were usually inhabit the upper respiratory tract and included asymptomatic children in different settings (Sulikowska et al., 2004). Additionally, *Streptococcus pneumoniae* and *Streptococcus anginosus* were the most common in children, which was enriched in the URTIs patients and consistent with the age-specific tendencies observed in a comprehensive surveillance research (Cao et al., 2025). Specifically, *Corynebacterium striatum* was reported detected in RTIs patients was in line with our previous study (Dong et al., 2024). It has been reported that *Acinetobacter baumannii* is often associated with hospital-acquired infections (Nguyen & Joshi, 2021), which was enriched in the LRTIs patients. Meanwhile, the fungi like *Candida albicans*, and viruses such as Epstein Barr virus and herpesvirus seem to play a more prominent role in LRTIs patients. The absence of a healthy control group precludes distinguishing colonization from infection. Many detected organisms, such as *S. pneumoniae* and *H. influenzae*, can be commensals in the upper respiratory tract but may become pathogenic under certain conditions (*e.g.*, viral coinfection, immune compromise) (Diaz-Diaz et al., 2022). Future multi-center, prospective studies with healthy controls and detailed clinical metadata are needed to validate these findings and explore causality.

The ARGs profiles observed in this study correlate with known resistance patterns in respiratory pathogens. In the RTIs population, the range of ARGs mainly include those associated with MLS, multi-drug, and beta-lactams antibiotics. This finding aligns with previous research on respiratory specimens from patients suffering from chronic obstructive pulmonary disease (COPD) and infectious diseases (Janjua et al., 2021). Notably, the gut microbiome predominantly harbors ARGs related to tetracyclines, MLS, and beta-lactams antibiotics (Pan et al., 2022). Such a disparity likely reflects the differences in microbial communities at various body sites and/or the variations in antibiotic exposure. Among common respiratory tract microbies, compared to other species, *Streptococcus pneumoniae* carries more ARGs, leading to enhanced resistance to MLS and aminoglycoside antibiotics. Notably, ARGs in the URTIs microbiome may be transferred among the resident microorganisms. The potential for ARGs transfer between commensal and pathogenic members of the respiratory microbiota represents an important area for future investigation.

We observed that tNGS can simultaneously identify pathogens and ARGs, offering advantages over culture-based methods. Our choice of tNGS over untargeted metagenomics was motivated by clinical applicability, cost-efficiency, and standardized validation for respiratory pathogen detection (Li et al., 2025). However, we acknowledge that primer-dependent amplification introduces detection bias and that the 199-pathogen panel may miss emerging or rare pathogens. The high concordance between our findings and established respiratory microbiology suggests that panel limitations did not substantially compromise biological conclusions, but clinicians should interpret negative results with awareness of panel scope. When dealing with different infection sites, it is advisable to utilize tNGS panels that encompass the relevant pathogen spectrum. For example, there are dedicated panels for pulmonary infections (Chen et al., 2024; Yang et al., 2025). The targeted pathogen spectrum should incorporate pathogens that are hard to culture and rare pathogens associated with the syndrome, while excluding microorganisms that are clinically insignificant for that specific site (Zhang et al., 2024). For patients with non-severe conditions, when conventional microbiological tests are unable to identify pathogens, tNGS is recommended.

The Random Forest model highlights that distinct microbial consortia are associated with URTIs and LRTIs. While infection site is determined clinically, the identification of these consortia, particularly when combined with ARGs profiles, may inform empirical antibiotic selection. For example, the high prevalence of beta-lactam resistance in LRTIs suggests that empiric therapy may require broader-spectrum agents or combination regimens, whereas URTIs-associated consortia may be more amenable to narrower-spectrum antibiotics.

There exist some limitations. First, the tNGS panel is limited to predefined targets, potentially missing emerging or unexpected pathogens. These limitations were mitigated by regular database updates and acknowledgment of panel constraints in data interpretation. Second, this study lacked a healthy control group, limiting our ability to distinguish pathogenic colonization from commensal microbiota. All interpretations regarding enrichment or differences are relative between URTIs and LRTIs patient groups rather than absolute deviations from healthy states. The retrospective nature precludes standardized sampling protocols and outcome tracking. We cannot establish temporal relationships between microbiome composition and disease progression, nor can we validate that tNGS results influenced clinical decision-making or patient outcomes. Third, potential confounders, including age, sex, sampling method, disease severity, prior antibiotic exposure, and care setting, may influence microbiome and ARGs profiles. Findings may reflect local epidemiological patterns, antibiotic prescribing practices, and patient demographics specific to our institution, limiting generalizability without multi-center validation.

## Conclusions

This research retrospective cohort study reveals that LRTIs having greater microbial diversity, and higher abundance of beta-lactam and multidrug resistance genes compared to URTIs. Moreover, tNGS is capable of quickly and accurately monitoring the emergence and dissemination of respiratory tract pathogens, ARGs, and virulence factors. While these findings advance understanding of site-specific microbial ecology in respiratory infections, the absence of healthy controls and outcome validation limits conclusions regarding diagnostic utility. Prospective multi-center studies with standardized protocols and clinical correlation are required to establish the clinical applicability of these observations.

## Supplemental Information

10.7717/peerj.21615/supp-1 Supplemental Information 1 R and RF code

10.7717/peerj.21615/supp-2 Supplemental Information 2 The pathogens detected in each patient with LRTIs and URTIs by the tNGS

10.7717/peerj.21615/supp-3 Supplemental Information 3 The ARGs detected in each patient with LRTIs and URTIs by the tNGS

10.7717/peerj.21615/supp-4 Supplemental Information 4 Patient information

10.7717/peerj.21615/supp-5 Supplemental Information 5 (A) The ARGs detected in different age group. (B) The ARGs detected in different gender group

## Additional Information and Declarations

### Data Availability

The following information was supplied regarding data availability: The data and code are available in the Supplemental Files. The raw sequence data are available at NCBI: PRJNA1313660.
