# Supplementary material for: Insights into microbiome and ARGs diversity in patients with upper and lower respiratory tract infections by targeted next-generation sequencing
Source: PeerJ. 2026 Aug 31;14:e21615. doi: 10.7717/peerj.21615 (PMC13544115; doi:10.7717/peerj.21615)
Supplement: Supplementary file 1 [file peerj-14-21615-s005.pdf]

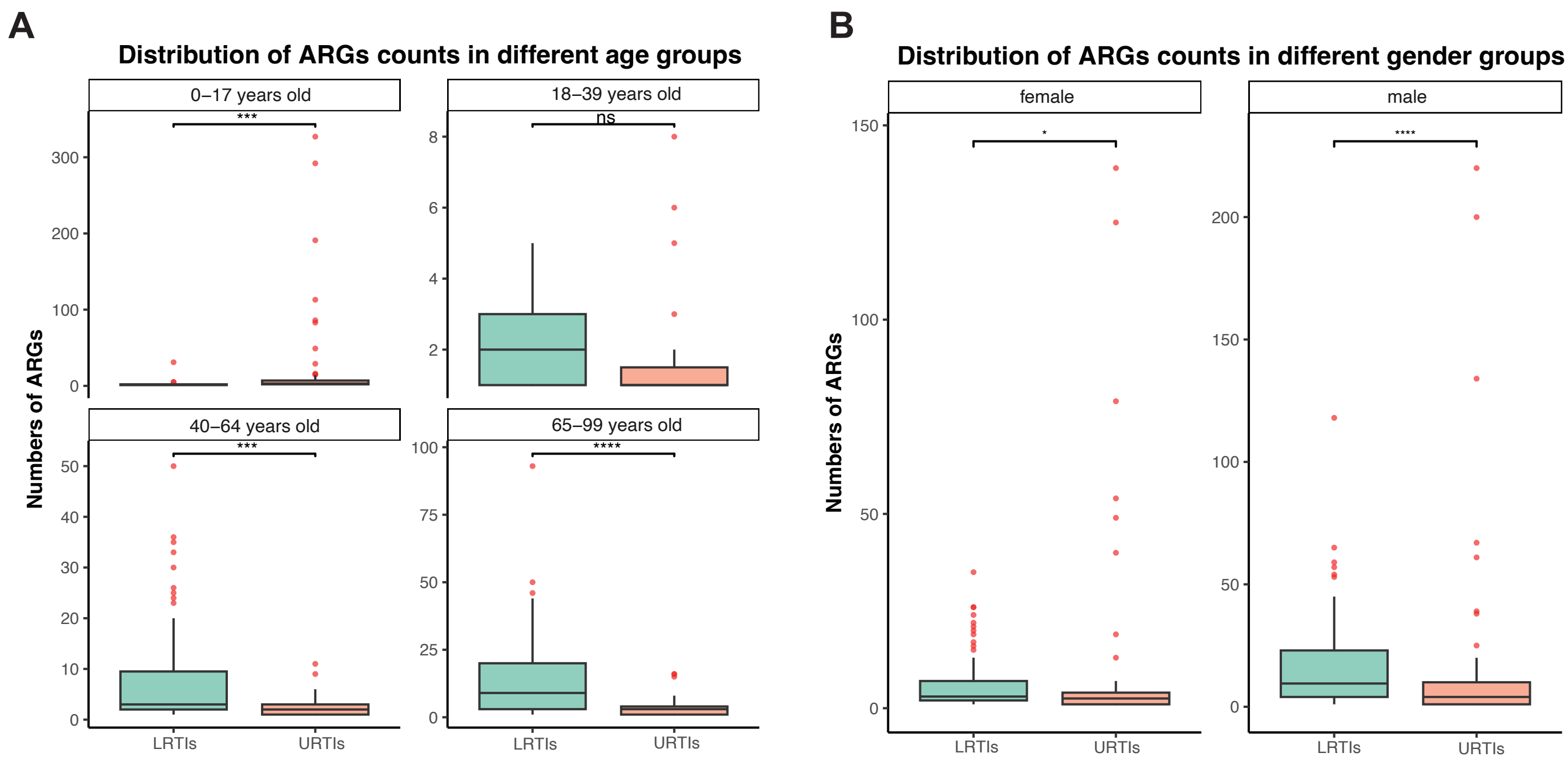

A
Distribution of ARGs counts in different age groups
0–17 years old
18–39 years old
40–64 years old
65–99 years old
***
ns
***
****
Numbers of ARGs
LRTIs
URTIs
B
Distribution of ARGs counts in different gender groups
female
male
*
****
Numbers of ARGs
LRTIs
URTIs
